# P01.48. Biomechanical responses to the mechanical characteristics of a spinal manipulation: effect of varying segmental contact site

**DOI:** 10.1186/1472-6882-12-S1-P48

**Published:** 2012-06-12

**Authors:** T Edgecombe, G Kawchuk, C Long, J Pickar

**Affiliations:** 1 University of Alberta, Edmonton, Canada; 2 Palmer Center for Chiropractic Research, Davenport, USA

## Purpose

Spinal manipulative therapy (SMT) is a common intervention used to treat low-back pain. Some prior investigations report decreased spinal stiffness following SMT; others report no effect. Given that the efficacy of many therapies (e.g. pharmaceuticals, injections) depend on choice of application site and outcome metric (e.g. fasting vs non-fasting blood sugar), potential explanations for mixed SMT results include variability in SMT application site and the method of computing stiffness. Based on these considerations, our goal was to determine the influence of SMT application site and stiffness computation on SMT-induced changes in spinal stiffness.

## Methods

In an anesthetized cat preparation (n=8), simulated SMT was delivered by a validated mechanical apparatus to the intact lumbar spine at 4 sites: L_6_ spinous process, left L_6_ lamina, left L_6_ mammillary process, and L_7_ spinous process. To obtain stiffness data, the apparatus slowly displaced the L_6_ spinous process to 16N; force and displacement were recorded continuously. Three metrics were calculated from the resulting force-displacement curve: Terminal Instantaneous Stiffness (TIS, stiffness at the end point of the curve), k (average stiffness), and Regional Stiffness (RS, average stiffness in each 10% interval of the curve). SMT-induced changes in each metric were determined for each application site using an ANOVA model controlling for SMT presentation order.

## Results

SMT applied at the L_6_ spinous decreased TIS (-0.48N/mm [-0.86, -0.09] upper, lower 95%CI). SMT applied at the L_6_ lamina also decreased TIS (-0.44N/mm; [-0.82, -0.05]). SMT applied to the L_6_ spinous increased k (0.44N/mm, [-0.01, 088]). SMT applied at L_6_ spinous process and L_6_ lamina decreased RS during some, but not all intervals.

## Conclusion

These results suggest that previous reports on SMT’s effect on spinal stiffness may be influenced by the choice of SMT application site and stiffness metric.

